# Decision Support Capabilities of Telemedicine in Emergency Prehospital Care: Systematic Review

**DOI:** 10.2196/18959

**Published:** 2020-12-08

**Authors:** Yesul Kim, Christopher Groombridge, Lorena Romero, Steven Clare, Mark Christopher Fitzgerald

**Affiliations:** 1 National Trauma Research Institute Melbourne Australia; 2 Monash University Melbourne Australia; 3 Trauma Services Alfred Health Melbourne Australia; 4 The Ian Potter Library Alfred Health Melbourne Australia

**Keywords:** telemedicine, decision support, emergency, resuscitation

## Abstract

**Background:**

Telemedicine offers a unique opportunity to improve coordination and administration for urgent patient care remotely. In an emergency setting, it has been used to support first responders by providing telephone or video consultation with specialists at hospitals and through the exchange of prehospital patient information. This technological solution is evolving rapidly, yet there is a concern that it is being implemented without a demonstrated clinical need and effectiveness as well as without a thorough economic evaluation.

**Objective:**

Our objective is to systematically review whether the clinical outcomes achieved, as reported in the literature, favor telemedicine decision support for medical interventions during prehospital care.

**Methods:**

This systematic review included peer-reviewed journal articles. Searches of 7 databases and relevant reviews were conducted. Eligibility criteria consisted of studies that covered telemedicine as data- and information-sharing and two-way teleconsultation platforms, with the objective of supporting medical decisions (eg, diagnosis, treatment, and receiving hospital decision) in a prehospital emergency setting. Simulation studies and studies that included pediatric populations were excluded. The procedures in this review followed the PRISMA (Preferred Reporting Items for Systematic Reviews and Meta-Analyses) statement. The Risk Of Bias In Non-randomised Studies–of Interventions (ROBINS-I) tool was used for the assessment of risk of bias. The results were synthesized based on predefined aspects of medical decisions that are made in a prehospital setting, which include diagnostic decision support, receiving facility decisions, and medical directions for treatment. All data extractions were done by at least two reviewers independently.

**Results:**

Out of 42 full-text reviews, 7 were found eligible. Diagnostic support and medical direction and decision for treatments were often reported. A key finding of this review was the high agreement between prehospital diagnoses via telemedicine and final in-hospital diagnoses, as supported by quantitative evidence. However, a majority of the articles described the clinical value of having access to remote experts without robust quantitative data. Most telemedicine solutions were evaluated within a feasibility or short-term preliminary study. In general, the results were positive for telemedicine use; however, biases, due to preintervention confounding factors and a lack of documentation on quality assurance and protocol for telemedicine activation, make it difficult to determine the direct effect on patient outcomes.

**Conclusions:**

The information-sharing capacity of telemedicine enables access to remote experts to support medical decision making on scene or in prolonged field care. The influence of human and technology factors on patient care is poorly understood and documented.

## Introduction

Effective communication has a central role in emergency care and trauma management [1-3]. Team coordination has been enhanced by information technology, which influences the way we work and interact. As an example, telephone consultation requests for remote experts are often made for complex patient scenarios between Level 1 trauma centers and rural hospitals [4]. The advent of portable devices, allowing real-time data and image transfer, has been shown to assist with making diagnoses in stroke and burns [5,6]. Heads-up-display (HUD) devices are being trialed in neurosurgical and orthopedic theaters for remote surgical guidance and vital signs monitoring [7,8]. Similarly, greater network coverage and processing and data speeds have increased the utility of smartphones and wireless devices for prehospital emergency medicine. In recent simulation and pilot studies, remote experts were able to support paramedics, or emergency medical technicians (EMTs), based on relayed data via telemedicine [9-11]. In this review, the term EMT will be used broadly to include all first responders providing medical care for the injured and/or ill.

*Telemedicine* is defined by the World Health Organization as the use of “Modern information and communication technologies (ICTs), such as computers, the Internet, and cell phones...to improve patient outcomes by increasing access to care and medical information” [12].

For the purpose of this review, the term telemedicine will cover ICT-facilitated two-way teleconsultation and data sharing between prehospital and hospital virtual hubs. Although various terms and methods define telemedicine, the overarching purpose of facilitating prehospital patient care and communication to the receiving hospital remains consistent across existing trials.

Prehospital resuscitation is delivered in a complex environment where responders make decisions under short time frames and with limited information. Factors such as a patient’s illness severity, ambient noise levels, fatigue, and stress can hinder decision making [13]. Various checklists and protocols have been designed, targeting out-of-hospital decision making. Over the years, computerized clinical decision support systems have also been implemented for in-hospital critical care and for disaster or combat and casualty decision making to provide teams with safety alerts, protocols, and diagnostic support for trauma management [14-17]. However, many fail to consider unstructured situations, technology resistance, and logistical support issues that may hinder reliable outcomes [18,19].

Research is limited on the decision-making mechanisms EMTs use at individual and organizational levels. It is unknown whether the currently available training programs and checklists mitigate human factors or environmental stressors that impact one’s decision-making processes [13]. A small number of studies investigating the decision-making processes of first responders show that they make use of intuitive reasoning, whereby their past experiences and first impressions on scene make the most impact, rather than an application of specific decision aids [20,21]. Importantly, information that cannot be assessed rapidly by visual or auditory scan (eg, blood pressure, heart rate, respiratory rate, and nonobvious anatomic injuries) is generally not part of a primary triage decision-making process [21]. For complex scenarios, a more deductive analytic method is used, with greater attention given to the available data [21].

In addition to the complexity of scenarios, prehospital care providers are now faced with making decisions over different patient care options. EMTs vary in the level of care they can provide [22]. Around the world, many services focus on rapid transport to hospital, thereby de-emphasizing interventions in the field, whereas in certain locations, specialized medical teams are able to undertake highly advanced interventions, such as extracorporeal membrane oxygenation [23,24]. Therefore, decisions over the course of prehospital care are made in the context of organizational constraints, culture, patient acuity, and evolving demands of EMTs.

In its simplest form, telephone consultation between EMTs and remote experts has been recognized as a beneficial tool [25,26], but the impact of telemedicine on clinical practice is poorly described. Understanding the medical decisions that are associated with telemedicine use as an intervention may have meaningful implications for training, protocols, and operations. As a first step, this systematic review aims to examine the evidence for telemedicine use with medical decisions made during prehospital care. Secondly, this review aims to examine whether telemedicine use contributes to decisions being made and whether it is associated with patient benefit.

## Methods

The methods in this review followed the PRISMA (Preferred Reporting Items for Systematic Reviews and Meta-Analyses) statement [27].

### Data Sources and Searches

The search strategy aimed to find published studies in 7 databases: MEDLINE, Embase, BIOSIS Previews, Emcare, PsycINFO, Scopus, and Web of Science. After an initial search for articles in MEDLINE and Embase, an analysis of the text words contained in the titles, abstracts, and index terms used to describe these articles was conducted. A second search using all identified keyword terms was then undertaken from database inception to March 29, 2019, across all 7 databases. Studies published in English were considered for inclusion in this review. The final searches were based on the MEDLINE search strategy (see Multimedia Appendix 1). Searches were adapted as appropriate to the specifications of all databases. Hand searching and reference checking of citations and reference lists were also undertaken. Authors of relevant studies were contacted if insufficient data were published.

### Study Selection

Articles on the implementation of telemedicine comprised of bidirectional communication via any device, with or without image- or data-sharing capabilities, in a prehospital setting for emergency and/or trauma cases were of interest. The following selection criteria were applied: (1) Population: patients attended by EMTs, (2) Intervention: telemedicine, (3) Outcomes: medical decisions made (eg, diagnosis and treatment), receiving facility decision, and any other clinical judgment, and (4) Setting: prehospital emergency.

The exclusion criteria were as follows: (1) the study included a pediatric population, (2) the study included a nonemergency setting, (3) the paper was only available as an abstract or poster, (4) the study included simulated trials, (5) the paper was a case study, editorial, dissertation, protocol, or review, and (6) the study was conducted purely for evaluating the feasibility and effectiveness of a technology. Studies including the pediatric population were excluded from this systematic review due to the incident rates associated with transportation via emergency services. Emergency medical transport use for pediatric cases has been reported at around 5%-10% [28,29]. Furthermore, medical interventions on children may require consent from their parents. Given the differences in incidence levels and medical interventions, this study focused on the telemedicine implementation for the adult population only.

Simulation trials have previously been included in systematic reviews on telemedicine applications in a prehospital setting [30-33]. This systematic review intended to examine telemedicine use in real-life scenarios to better understand its clinical value and limitations, as it is yet to be embedded into widespread routine use. We included studies that did not have a comparator or control group. Although this limited the evidence examined, it provides a comprehensive overview of telemedicine outcomes under actual emergency circumstances.

### Data Extraction

First, one reviewer (YK) independently evaluated the titles and abstracts of all records identified in the initial databases search. Two reviewers (YK and CG) then assessed the eligible full-text articles and, if necessary, discussed their suitability. Additionally, references from reviews and journal articles were screened by one of the reviewers. Disagreements on questions of eligibility were resolved through discussion and none of the articles required an escalation to a third reviewer (MF).

Once a decision was reached, in line with the inclusion criteria, the selected full texts were reviewed by YK and CG. Data were extracted regarding the medical emergency category, the telemedicine platform, and each platform’s associated decision outcomes. Any medical decisions or clinical judgments made as an outcome of the telemedicine intervention by the journal authors were considered. Technical performance, with regard to device failure and network issues, was noted if it interfered with the delivery of patient care. Study characteristics were also obtained, including sample size, trial location, and study design.

### Risk-of-Bias Assessment

After individual assessments, two reviewers discussed and agreed upon the quality assessment of each article. In order to cater to the heterogeneity in study design, the Risk Of Bias In Non-randomised Studies–of Interventions (ROBINS-I) tool [34] was used to evaluate the methodological rigor in 6 studies. This risk assessment tool was deemed appropriate, as it is particularly concerned with evaluating the effectiveness or safety of an intervention from studies that did not use randomization in allocation.

This tool covers 7 domains of bias that can be introduced from using nonrandomization. Two preintervention biases are particularly of concern in nonrandomized studies. Confounding factors considered for telemedicine intervention were the experience level of EMTs, injury severity for all emergencies, and intervention awareness. Failing to control for such confounders may reduce the comparability between the intervention and control groups. Selection bias was considered a risk whenever eligible participants were excluded in a way that could lead to an association between telemedicine and outcomes. An example of a confounder is if no explanation was provided for how telemedicine was implemented for the study group as compared to the controls during the trial period. Classification bias of interventions at the time of the study was related to the information about the delivery and structure of telemedicine, patient medical records, and any organizational records obtained. Problems of recall bias and subjective opinions can be avoided if voice communication was recorded and assessed by persons not involved in resuscitation and if the medical records assessed were a part of routine care prior to the commencement of the study.

Biases can also arise when there are differences in the care provided, between the study and control groups, due to the awareness of expectations or outcomes of the study; most studies cannot be blinded when using telemedicine. Importantly for telemedicine, performance bias and adherence may be factors that were of interest under risk assessment. Biases during the postintervention phase relate to data handling, particularly the measurement of outcomes; how the results were reported; and how missing data were handled. In particular, we noted whether there was an outcome assessment measure that was applied across cases and whether the outcome could easily be influenced by the assessor being aware of telemedicine being implemented.

One of the included studies was a review of cases and did not have enough information to be assessed by ROBINS-I. This particular study was assessed based on confounders, measurement of outcomes, missing data, and reporting biases. The two investigators (YK and CG) discussed the possible biases and the evidence in support of the decisions.

### Data Analysis

Study characteristics were clinically heterogeneous. For example, there was no consistent dependent variable that could be aggregated. As previously mentioned, a standard method for implementing telemedicine or measuring relevant outcomes is yet to exist. Thus, it was not possible to aggregate the findings in a quantitative meta-analysis. Instead, the results were synthesized based on patterns and themes that were in line with the aim of this systematic review. Decisions made using telemedicine solutions—diagnostic decision support, receiving facility decisions, and medical directions for treatment—were examined for primary outcomes of interest. Lastly, mortality, adverse events, and technical challenges were noted. The challenges not only reflected device or network-related issues, but also the effect of telemedicine on individual workflow, clinical governance, and overall organizational support.

## Results

### Overview

**Figure 1:**
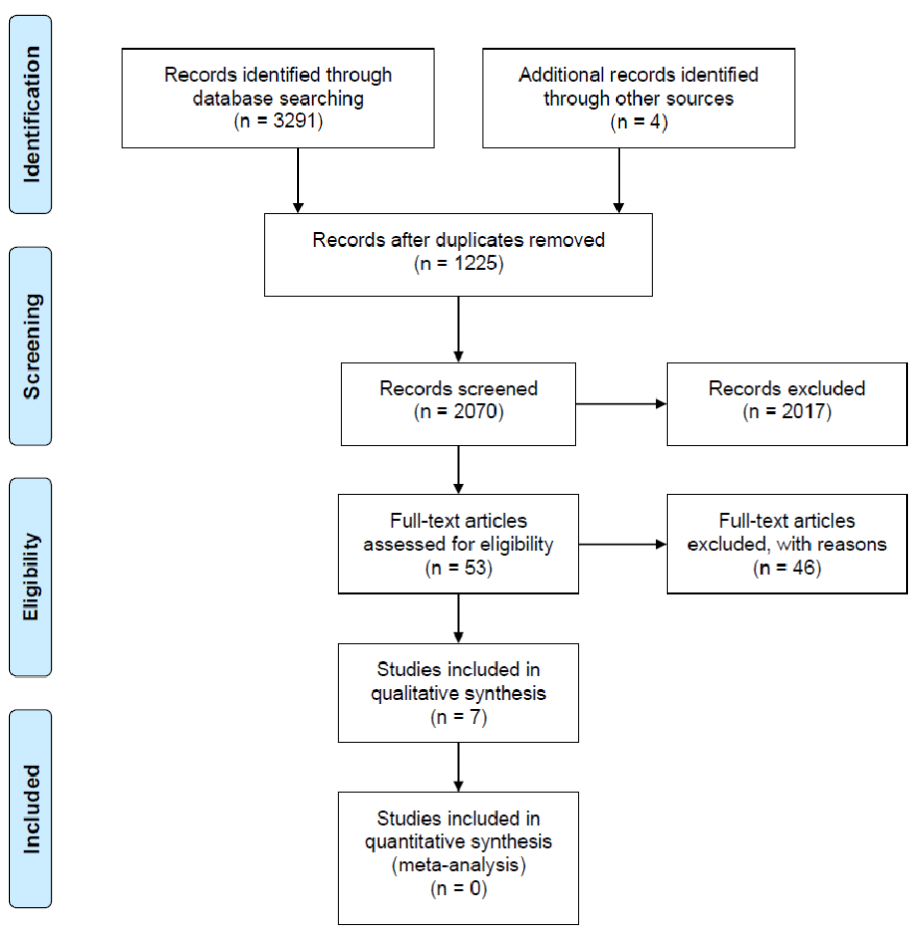
PRISMA (Preferred Reporting Items for Systematic Reviews and Meta-Analyses) flowchart.

The initial search on 7 databases yielded 3291 records, where 1225 duplicates were identified. Based on the inclusion criteria, 2017 articles were excluded and the remaining 49 articles proceeded to full-text examination. Of those, 42 studies were excluded due to the setting and/or outcomes of interest. Figure 1 represents the search results as a PRISMA flowchart.

### Study Characteristics

Table S1 in Multimedia Appendix 2 shows the study characteristics of 7 selected studies, including the details of telemedicine technologies used. There were only a small number of papers identified that examine telemedicine as an intervention in the real-world setting. This suggests that despite the increasing popularity of this tool, research has been limited to simulated scenarios or halted at pilot phases. Furthermore, the methodologies reflect the nascent nature of this research field, where no framework or systematic approach exists for (1) telemedicine implementation or (2) assessment measures to evaluate its impact on clinical outcomes.

Surprisingly, none of the studies incorporated an HUD device as a component of the telemedicine system. In most interventions, smartphones or accessory equipment (eg, Bluetooth headset) enabled voice communication to a remote expert. Three studies focused their findings on two-way communication, via telephone, as the main method to deliver expert guidance [35-37]. These studies often relayed patient vital signs and other necessary information verbally, in turn gaining medical direction. For projects hoping to expand the use of telemedicine into routine use, ambulances were equipped with built-in platforms for audio and video communication, real-time vital sign transmission systems using portable devices [35,38-40]. Details about the technology used for voice communications were not provided in Dulou et al’s study [37]; the authors primarily based their findings on surveys and transcribed data from after-action reviews (see Table S1 in Multimedia Appendix 2).

### Bias Assessment

The risk of bias was assessed as moderate in the majority of studies, as they were nonrandomized and prospective in design (see Table S2 in Multimedia Appendix 2). Due to the type of intervention and the pre-exposure and pretraining that may be required, confounder bias was common among the studies. Examples include severity of patient illness, knowledge of intervention and performance assessment, and skill level of the paramedic. As most studies aimed to correlate telemedicine use with positive outcomes, we cannot exclude the possibility that only the most capable EMTs, clinically and technologically, were chosen for a preliminary *feasibility* phase, limiting the study’s real-world applicability and validity. Information on missing data was unclear or had moderate bias in 5 studies [35,37-39,41]. Intervention bias was difficult to assess, as there appeared to be no systematic way of implementing teleconsultation and the medical direction given by a remote expert. There were trials [35,38,40] where the outcome measure was vulnerable to subjectivity, as the knowledge of telemedicine intervention could influence the interpretation of its clinical value (see Tables S2 and S3 in Multimedia Appendix 2).

### Decisions Made From Using Telemedicine Solutions

Three categories under decision support were collated by the authors: diagnostic decision support, receiving facility and disposition decisions, and medical direction for prehospital treatments.

#### Diagnostic Decision Support

Remote expert consultation, facilitated by patient vital signs and image transfers, was useful in making accurate prehospital diagnoses. For example, evidence of diagnostic decision support was present for the majority of emergency cases where the telemedicine system was available, of which about 23% were trauma related [39]. Diagnoses were not only made using telemedicine but were made accurately, indicated by a high agreement between prehospital diagnoses via telemedicine and final in-hospital diagnoses (see κ statistics in Table S3 in Multimedia Appendix 2) [40].

Notably, usefulness of diagnostic support did not differ significantly in relation to the method of communication used. In a study comparing methods used for medical direction, opinions of consulting physicians equally favored telephone calls (mean score of 4 on a 5-point Likert scale; n=107) and full telemedicine capability with real-time data transferring and video-enabled communication (mean score of 3 on a 5-point Likert scale; n=32) [35]. EMTs also found telephone calls sufficient to relay patient status compared to using a real-time data transfer option. These findings could be explained by the introduction of added workload when using telemedicine in comparison to the simplicity of requesting a telephone consultation; however, neither a qualitative nor a quantitative explanation was offered in the study. Nevertheless, other studies [38] have reported the value of image transmission in providing an accurate situational assessment for a case discussion with remote experts.

#### Receiving Facility Decisions

The clinical significance of selecting an appropriate hospital destination through remote expert support is highlighted by Kim and colleagues’ [41] findings. In countries where the local ambulance service is less integrated into a larger, state or national, emergency medical system, such as Korea, the receiving hospital is often determined by patients and/or their family members. Consequently, patients with mild medical conditions are often transferred to larger hospitals, overcrowding their emergency rooms, intensive care units, and general wards [41]. A real-time telemetry system (RTS) facilitated hospital destination decisions by relaying more patient information to the hospital and understanding their resource availability. Using an RTS, the destination decisions were tailored to individual patients based on medical indications [41].

Furthermore, early communication activated team responses, treatment plan decisions, and preparation for patient arrival [40]. Similarly, Bergrath et al [39] observed that in-hospital treatments could be prearranged based on prehospital notification, which ultimately reduced in-hospital time intervals and improved patient outcomes. However, such secondary outcomes were not recorded nor observed consistently across different cohorts of emergencies, which included trauma; therefore, it is difficult to assume generalizability for these findings given that patients vary in mechanisms of injury and severity.

#### Medical Direction for Prehospital Treatment Decisions

Medical direction for triage, treatment, and/or surgical guidance was present in all of the selected studies. These findings, however, should be interpreted in light of missing data, potential confounders, and bias in measurement of outcomes. Decision support for prehospital treatment was especially relevant for EMTs with a limited scope of skills under a regional or organizational jurisdiction. As evidenced in Germany, telemedicine technology enabled EMTs to start intravenous medication administration prior to hospital arrival with tele-doctor presence [39].

Joint treatment decisions between an on-scene first responder and remote experts were also enabled by in-built telemedicine systems. For example, the transmission of prehospital electrocardiograms and early involvement of the remote specialist ultimately shortened the door-to-balloon time in hospital [38]. The authors emphasized that such successes were made possible by an organizational approach to telemedicine with a standardized process and protocols to follow. Bergrath and colleagues [38] further found that voice communication, vital signs, and image transmission facilitated remote experts in better assessing the situation, which was important for subsequent medical directions.

Kim et al [41] developed the RTS by which patients’ vital signs are transmitted to an emergency medical information center. The mean prehospital treatment time at the scene for the telemedicine group was shorter than for the control group for all patients, irrespective of the injury severity. Importantly, the authors interpreted that telemedicine was useful for receiving guidance not only on basic life support but also with advanced life-support interventions.

In particular, when there may be an experience-acquisition gap, for example, in an austere combat casualty setting, telemedicine played an invaluable role. In 14.8% of cases, telemedicine was necessary for patient management during prolonged field care. Prolonged field care requires ongoing assessment, interpreting vital trends over time, and identifying early decompensation, all of which are infrequently encountered by many prehospital providers in metropolitan areas. In such scenarios, guidance for clinical decisions, including differential diagnoses and treatment plans, were available from remote experts for frontline medics [36]. In warzone areas—Africa, Central Europe, and Afghanistan—surgical coaching and advice regarding further patient management was also made possible by telemedicine [37]. These findings are often descriptive and lack details on specific clinical outcomes. Nevertheless, the authors highlight that in its most basic form, a simple telephone consultation is highly effective and easily adopted by first responders in the military setting.

While telemedicine appears to facilitate prehospital treatment decisions, confounders (eg, paramedic skill level and patient severity) and an absence of randomized controls in many studies may overestimate a true relationship. A recent feasibility study conducted by Yperzeele and colleagues [40] reported that over 90% of teleconsultations were clinically useful, although the study did not allow the interventions to be decided by remote experts. It is difficult to understand how the remote experts were able to influence treatment plans delivered by the EMTs. Cho et al [35] used negative controls in order to demonstrate the usability of an ambulance-embedded telemedicine solution over routine use of telephone calls for making medical decisions. When comparing a telemedicine system to telephone consultation only, the authors concluded that there were no advantages in incorporating a full telemedicine capability (eg, real-time data transfer) in the ambulances. The introduction of telemedicine significantly reduced consultations for on-scene treatment, possibly due to the friction cost of using an unfamiliar system. Importantly, the case load and patient presentations were similar before and after telemedicine implementation, suggesting that case load and patient acuity were not responsible for hindering telemedicine adoption.

Overall, the results suggest that telemedicine is useful, especially in challenging and unpredictable environments. However, the methodologies used and the study characteristics make findings inconclusive. Replication is required in larger clinical trials with a randomized control group in order to better understand the usability, adoption, and reliability of telemedicine.

### Mortality, Hospital Stay, Adverse Events, and Technical Challenges

No adverse events or safety concerns were raised during or after telemedicine intervention [39,40]. Yperzeele and colleagues [40] also reported no patient complaints or refusals for telemedicine use. Kim et al’s [41] study was the only one to compare mortality between the study group and controls. There was no statistically significant difference between the RTS group and the control group in mortality (*P*=.45) and length of stay in the emergency department (*P*=.82). Overall mortality was examined by DeSoucy et al [36]; however, the effects of telemedicine were not considered for statistical analyses. Cho et al [35] and Bergrath et al [38] did not report on adverse events, safety, or mortality.

### Telemedicine Technology Adoption and Challenges

In terms of perceived user satisfaction with telemedicine solutions, Yperzeele et al [40] measured general acceptance of the system, which was high for both teleconsultants and nurses seeking advice. These findings were determined from high rates of system activation (75.4%) and a Likert scale intended to rate user friendliness. In another study, technology adoption was measured against overall quality and clinical value, where 10 missions using video transmission were deemed to be of major clinical value [38]. Nevertheless, photos appeared to be preferred by remote experts when compared to videos, due to inconsistent transmission quality. Similarly, no significant advantage was found between telephones or video-streaming features to enable two-way communication [35].

With regard to technical challenges observed, network failures and limited processing power for video transmission and streaming most frequently disrupted telemedicine use [35,38-40]. Notably, technical issues were more difficult to overcome without protocols and overall clinical governance in place [35]. This meant that more effort was required of frontline personnel to resolve technical issues, with a consequential increase in cognitive load and a disruption of workflow.

## Discussion

This systematic review examined whether clinical decisions are associated with telemedicine use in a prehospital emergency medical setting. To date, feasibility studies, simulated randomized controlled trials [9,26], and systematic reviews [30,32,42] on telemedicine fail to adequately address the role of decision making in patient outcomes. It is difficult to access information on (1) how telemedicine has been implemented and if any protocols exist and (2) measurements of clinical outcomes before versus after telemedicine implementation. Overall, studies investigating the effectiveness of telemedicine are heterogeneous and their findings cannot be aggregated quantitatively. The authors’ interpretations of findings are typically based on observations, descriptive statistics, and surveys collected from the end users. Such methods contain inherent confounding bias, and with a small sample, often without adequate comparators, they present unavoidable challenges in delivering translatable outcomes.

Despite moderate biases present in most studies, the authors are in agreement that if employed appropriately (eg, specific patient cohort type and device and network reliability) and with organizational support, telemedicine provides invaluable access to decision support that is otherwise unobtainable. This includes support for making prehospital diagnoses [38-40], support for receiving facility decisions [38,39,41], and medical procedure guidance [35,38,41]. Medical directions appear to have the most impact in settings where EMTs require a doctor’s input for medication administration and/or procedures [39,41]. The enhanced communication between EMTs and hospital medical staff, augmented by data and image transmission, all substantiate the use of telemedicine to improve the quality of continuing care.

Importantly, telemedicine may be most useful when there is an experience-acquisition gap, as there will inevitably be complex presentations or unanticipated complications. Furthermore, in rural emergency settings, the duration of patient retrieval and initial patient care can be longer [36,41]. Such scenarios may benefit from teleconsultation for ongoing monitoring and feedback on treatment plans based on real-time vital sign updates. The association between prehospital vital signs, injury severity, and in-hospital clinical outcomes highlight the importance of making prehospital data available for the severely injured [43-46]. In reality, a few vital sign data points are usually transmitted via telephone before, during, or after ambulance transfer, and whether this is adequate or not is yet to be determined.

**Figure 2:**
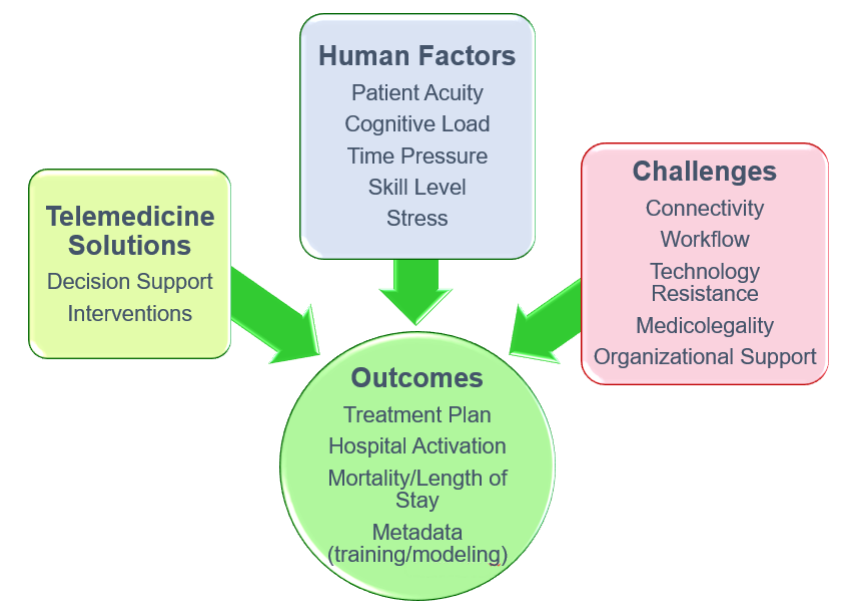
A network of factors influencing outcomes related to hospital operation and patient care.

Unfortunately, the evidence for potential downstream effects is anecdotal and not yet described in existing literature. Authors [35,36,41] identified by this systematic review anticipate downstream effects, discussed below in this section, with larger trials and further research and development on telemedicine implementation. Preliminarily, it appears that the vital sign trends received by the hospital have implications for increased readiness in patient reception, with appropriate equipment and blood products and team formation [35]. Real-time data sharing and remote expert consultation may allow for the preparation of tailored treatments and interventions prior to the patient’s arrival at hospital. Shortened on-scene times may also be achieved with teleconsultation [39], with the possibility of reduction in overall transfer time [41]. Finally, improved disposition decisions were often reported, which may achieve significant cost savings through better use of emergency resources and a reduction in unnecessary secondary transfers. Figure 2 summarizes the interplay between the tasks faced by the paramedic, human factors, and telemedicine technology in influencing in-hospital and potential long-term outcomes.

Based on the findings of this systematic review, future research should develop standardized clinical and technical protocols for telemedicine implementation. Bergrath and colleagues [38] highlighted the importance of an organizational approach in overcoming technical issues and achieving technology adoption. A solution for connection issues may be easier to achieve than developing clinical protocols and user case scenarios to ensure safety and systematic implementation of the technology, especially in the event of connection failure. At the very least, these findings stress the need for technical issues to be resolved, with prioritized connection to the hospital team for data and image transfer for severely injured patients. Importantly, the EMTs’ workflow should be considered, such that real-time data transmission and remote expert interaction requires minimal cognitive load. To be effective, developed user case scenarios will need to target various patient presentations and paramedic experience levels and will need to account for human factors that may hinder one’s decision-making processes under stress [13]. With regard to decision support, the cooperation of physicians in providing structured, measurable feedback is useful in correlating specific clinical outcomes and audits.

This systematic review has potential limitations, mostly stemming from the inclusion of nonrandomized studies. Specifically, these include difficulties in assessing, as well as having limited access to, a full data set and the risk of selective reporting in nonrandomized studies. The theoretical foundation for telemedicine solutions and the roles they play in *decision making* that are relevant to the aims of this review are also limited. In relation to the search strategy, the term *telemedicine* may have missed other decision support technological tools (eg, clinical decision support systems) with embedded remote expert communication and data-sharing capacity. Lastly, publication bias may exist for feasibility studies, as they are more likely to report positive outcomes in order to conduct subsequent clinical trials.

In conclusion, there is a role for telemedicine in supporting prehospital decision making for diagnoses, lifesaving interventions, and hospital destination. Although research in this area is in its infancy, further research into telemedicine as a tool for decision support, in real patient encounters, deserves more attention. As one of the authors highlights, “clearly, not all aspects of an emergency can be addressed by teleconsultation, *but in cases when a medical decision must be made*, it may provide a beneficial alternative” [39]. It is conceivable that telemedicine instills decision-making confidence in prehospital providers to commit to a treatment plan. An important function of the remote expert is to provide guidance when managing unanticipated events, such as during times of disaster or unprecedented virus outbreak [47,48]. In order to increase proficiency, accountability, and improved patient care, prehospital providers should consider using teleconsultations during training as well as in clinical practice.

## Appendix

Multimedia Appendix 1 MEDLINE search strategy.

Multimedia Appendix 2 Characteristics, risks of bias, and summary of findings of selected studies.

## Abbreviations

EMT: emergency medical technician
HUD: heads-up display
ICT: information and communication technology
PRISMA: Preferred Reporting Items for Systematic Reviews and Meta-Analyses
ROBINS-I: Risk Of Bias In Non-randomised Studies–of Interventions
RTS: real-time telemetry system
