# Supplementary material for: Decision Support Capabilities of Telemedicine in Emergency Prehospital Care: Systematic Review
Source: J Med Internet Res. 2020 Dec 8;22(12):e18959. doi: 10.2196/18959 (PMC7755537; doi:10.2196/18959)
Supplement: Multimedia Appendix 1 [file jmir_v22i12e18959_app1.docx]

Database(s): **Ovid MEDLINE(R) and Epub Ahead of Print, In-Process & Other Non-Indexed Citations, Daily and Versions(R)**1946 to March 22, 2019 Search Strategy:

| **#** | **Searches** | **Results** |
| --- | --- | --- |
| 1 | "transportation of patients"/ or ambulances/ or air ambulances/ | 15708 |
| 2 | emergency medical services/ or emergency medical dispatch/ or emergency medical service communication systems/ | 41101 |
| 3 | emergency responders/ or emergency medical technicians/ or firefighters/ or police/ | 11455 |
| 4 | (paramedic* or prehospital or pre-hospital or ambulance* or emergency medical service* or emergency medical technician* or first responder* or 1st responder* or emergency responder* or field triage or emergency medical care or emergency care or firefighter* or fire-fighter* or rescue personnel or mountain rescue* or out-of-hospital or adult retrieval* or patient retrieval* or clinical transport* or field care* or field medicine* or helicopter*).mp. | 82903 |
| 5 | 1 or 2 or 3 or 4 | 92278 |
| 6 | telemedicine/ or remote consultation/ or teleradiology/ or Telemetry/ | 32530 |
| 7 | telecommunications/ or satellite communications/ or videoconferencing/ or wireless technology/ | 10163 |
| 8 | computers, handheld/ or smartphone/ or Cell Phone/ or Robotics/ | 32141 |
| 9 | computer communication networks/ or internet/ or internet access/ | 80257 |
| 10 | (Telemedic* or Teleconsult* or Telesonograph* or Telecare or Telecooperation or Telehealth or Telementor* or Telematic* or Telediagnos* or Teleintensive care or Teleecho* or Teleradiolog* or Teleultrasound or Telemetry or Telepresence or Telecommunication* or Teleconference*).mp. | 46420 |
| 11 | (Tele-medic* or Tele-consult* or Tele-sonograph* or Tele-care or Tele-cooperation or Tele-health or Tele-mentor* or Tele-matic* or Tele-diagnos* or Tele-intensive care or Tele-echo* or Tele-radiolog* or Tele-ultrasound or Tele-presence or Tele-communication* or Tele-conference*).mp. | 584 |
| 12 | ((image* or data or monitoring or picture* or vital parameter*) adj4 (transmi* or transfer*)).mp. | 20470 |
| 13 | (real-time adj (interactive or mobile or ultraso* or echo* or image* or vital data or medical or TS system* or monitoring or audio or video or guide* or guidance or remote or medical)).mp. | 11168 |
| 14 | (video adj2 (consult* or conferenc* or transmi*)).mp. | 1204 |
| 15 | ((Audio-video or audiovisual or audio-visual) adj (feed* or interface or communication or transmi*)).mp. | 196 |
| 16 | (stream* adj3 (live or ultraso* or audio or video or scan* or image*)).mp. | 906 |
| 17 | (videoconference* or videostream* or livestream*).mp. | 657 |
| 18 | (remote* adj3 (examination* or assessment* or video or just-in-time or control* or robotic or interpret* or support* or consult* or mentor* or guide* or guidance or observe* or direction)).mp. | 9560 |
| 19 | (communications coverage or broadband access or high speed broadband).mp. | 57 |
| 20 | ((mobile or cellphone or wifi or "wi-fi" or internet) adj (hotspot or connecti* or network*)).mp. | 1016 |
| 21 | ((transtelephonic or trans-telephonic) adj2 (communication* or transmi* or monitor*)).mp. | 191 |
| 22 | 6 or 7 or 8 or 9 or 10 or 11 or 12 or 13 or 14 or 15 or 16 or 17 or 18 or 19 or 20 or 21 | 187054 |
| 23 | 5 and 22 | 2362 |
| 24 | exp "Wounds and Injuries"/ | 859493 |
| 25 | (Wound* or injur* or rupture* or perforat* or penetrat* or fracture* or burn* or trauma* or postinjur* or posttrauma* or polytrauma* or multitrauma* or neurotrauma*).mp. | 1965087 |
| 26 | trauma centers/ or traumatology/ or multiple trauma/ | 23781 |
| 27 | trauma severity indices/ or abbreviated injury scale/ or glasgow coma scale/ or glasgow outcome scale/ or injury severity score/ | 31835 |
| 28 | Critical Illness/ | 25414 |
| 29 | accidents/ or accidents, home/ or accidents, occupational/ or accidents, traffic/ or near drowning/ | 77573 |
| 30 | exsanguination/ or (exsanguinat* or coagulopath*).mp. | 16419 |
| 31 | cerebral hemorrhage, traumatic/ or intracranial hemorrhage, traumatic/ or brain hemorrhage, traumatic/ or hematoma, epidural, cranial/ or hematoma, subdural/ or hematoma, subdural, acute/ or hematoma, subdural, chronic/ or hematoma, subdural, intracranial/ or subarachnoid hemorrhage, traumatic/ | 12129 |
| 32 | (stab or stabbing or stabbed or gunshot* or gun shot* or shotgun* or shot gun*).mp. | 31398 |
| 33 | ((blood* or bleed* or h*emo*rhag*) adj2 extravasation).mp. | 548 |
| 34 | ((epidural or subdural) adj (h*ematoma* or h*emo*rhag*)).mp. | 11869 |
| 35 | ((glasgow or rancho los amigos) adj2 (scale* or score*)).mp. | 18966 |
| 36 | 24 or 25 or 26 or 27 or 28 or 29 or 30 or 31 or 32 or 33 or 34 or 35 | 2181807 |
| 37 | 23 and 36 | 496 |
| 38 | ((head-up or heads-up or headup or headsup) adj6 (display* or visual* or navigat* or comput* or system* or technolog* or platform* or device* or symbol* or image* or imaging* or conformal* or nonconformal* or scene* or view* or project*)).mp. | 271 |
| 39 | ((head-mount* or headmount* or helmet-mount* or head-worn or headworn) adj (display* or visual* or navigat* or comput* or system* or technolog* or platform* or device* or symbol* or image* or imaging* or conformal* or nonconformal* or scene* or view* or project*)).mp. | 872 |
| 40 | ((face-mount* or face-worn) adj (display* or visual* or navigat* or comput* or system* or technolog* or platform* or device* or symbol* or image* or imaging* or conformal* or nonconformal* or scene* or view* or project*)).mp. | 8 |
| 41 | (wearable adj (display* or navigat* or comput* or system* or technolog* or platform*)).mp. | 1080 |
| 42 | ((head-up or heads-up or headup or headsup) adj (monitor* or technique* or method* or time* or task*)).mp. | 8 |
| 43 | ((windshield* or windscreen*) adj3 (display* or imag* or symbol* or digital* or project* or navigat* or comput* or technolog* or project*)).mp. | 8 |
| 44 | ((dashboard-mount* or eyeglass-mount* or smart glass*) adj3 (display* or imag* or symbol* or digital* or project* or navigat* or comput* or technolog* or project*)).mp. | 17 |
| 45 | ((near-eye or goggle) adj (display* or navigat* or comput* or system* or technolog* or platform*)).mp. | 56 |
| 46 | ((HUD or HUDs or HMD or HMDs or HWD or HWDs) adj (display* or visual* or navigat* or comput* or system* or technolog* or platform* or device* or symbol* or image* or imaging* or conformal* or nonconformal* or scene* or view* or project*)).mp. | 83 |
| 47 | ((augmented reality or virtual reality or augment* visual* or visual* augment*) adj (goggle* or glass* or screen* or display*)).mp. | 128 |
| 48 | google glass*.mp. | 151 |
| 49 | (((augmented reality or virtual reality or augment* visual* or visual* augment*) adj4 (display* or visual* or navigat* or comput* or system* or technolog* or platform* or device* or symbol* or image* or imaging* or conformal* or nonconformal* or scene* or view* or project*)) and ((computer-assisted or computer-aided or computer-guided or image-guided or video-assisted) adj3 (surg* or therap*))).mp. | 453 |
| 50 | data display.mp. | 7129 |
| 51 | 38 or 39 or 40 or 41 or 42 or 43 or 44 or 45 or 46 or 47 or 48 or 49 or 50 | 9845 |
| 52 | 5 and 36 and 51 | 12 |
| 53 | (Teletrauma or tele-trauma).mp. | 34 |
| 54 | 5 and 53 | 9 |
| 55 | 37 or 52 or 54 | 502 |
| 56 | limit 55 to english language | 461 |
