# Supplementary material for: Decision Support Capabilities of Telemedicine in Emergency Prehospital Care: Systematic Review
Source: J Med Internet Res. 2020 Dec 8;22(12):e18959. doi: 10.2196/18959 (PMC7755537; doi:10.2196/18959)
Supplement: Multimedia Appendix 2 [file jmir_v22i12e18959_app2.docx]

**Table S1.** Study characteristics.

| **1st Author** | **Year** | **Country** | **Study Design** | **Setting** | **Participants (treatment/control)** | | **Intervention (devices used for telemedicine)** | **Comparison** |
| --- | --- | --- | --- | --- | --- | --- | --- | --- |
|  |  |  |  |  |  |  |  |  |
| Bergrath, S. | 2011 | Germany | A prospective, observational study | Life-threatening emergencies | 157 | - | Bluetooth headset, portable data transmission unit | N/A |
|  |  |  |  |  |  |  |  |  |
| Bergrath, S. | 2013 | Germany | A prospective, observational study | Prehospital emergency | 35 | 296 | Smart phone, portable data transmission unit | Routine care |
|  |  |  |  |  | (8, Trauma) |  |  |  |
| Cho, S. J. | 2015 | South Korea | A retrospective cohort study | Prehospital emergency | 82 | 117 | M-KIOSK system (PhysioLab Inc., Busan, Korea) | Baseline control prior to intervention |
|  |  |  |  |  | (18, Trauma) | (26, Trauma) |  |  |
| DeSoucy, E. | 2017 | United States | A review of cases | Higher level of medical care with +4 hours prehospital time | 8 | - | Telephone (not specified) | N/A |
|  |  |  |  |  |  |  |  |  |
| Dulou, R. | 2010 | France | A descriptive study | Neurotrauma | 2 | - | Telephone (not specified), Mobile Neurosurgical unit | N/A |
|  |  |  |  |  |  |  |  |  |
| Kim, Y. K. | 2011 | South Korea | A retrospective cohort study | Prehospital emergency | 188 | 750 | Real-Time Telemetry System (RTS, Korea) | no RTS |
|  |  |  |  |  |  |  |  |  |
| Yperzeele, L. | 2014 | Belgium | A prospective, feasibility study | Prehospital emergency | 41 | 134 | PreSSUB 3.0 system (Brussels, Belgium) | Routine care |
|  |  |  |  |  | (4, serious trauma) |  |  |  |

**Table S2.** Risk assessment of selected studies for systematic review.

| **Study** | | **Pre-Intervention** | | **At Intervention** | **Post-Intervention** | | | | **Overall risk of Bias** |
| --- | --- | --- | --- | --- | --- | --- | --- | --- | --- |
| First Author | Year | Bias due to confounding | Bias in selection of participants into the study | Bias in classification of interventions | Bias due to deviations from intended interventions | Bias due to missing data | Bias in measurement of outcomes | Bias in selection of the reported results | low / moderate / serious / critical |
|  |  |  |  |  |  |  |  |  |  |
|  |  |  |  |  |  |  |  |  |  |
|  |  |  |  |  |  |  |  |  |  |
| Bergrath, S. | 2011 | Serious | Low | Low | Low | Moderate | Serious | Moderate | Moderate |
| Bergrath, S. | 2013 | Moderate | Serious | Low | Low | Not enough Info | Low | Low | Low-Moderate |
| Cho, S. J. | 2015 | Serious | Moderate | Low | Low | Moderate | Serious | Low | Moderate |
| Dulou, R. | 2010 | Serious | Low | Moderate | Low | Not enough Info | Not enough Info | Serious | Serious |
| Kim, Y. K. | 2011 | Serious | Not enough Info | Moderate | Moderate | Not enough Info | Low | Low | Moderate |
| Yperzeele, L. | 2014 | Moderate | Low | Low | Low | Low | Moderate | Low | Low |

**Table S3.** Summary of findings.

| **1st Author** | **Outcomes** | **Results** | **Study conclusions** | **Comments** |
| --- | --- | --- | --- | --- |
| Bergrath, S. | Receiving facility decision | 18% | Joint medical decisions made possible by VS transmission and voice commmunication. Clinical value of image transfer alone was rated as 'helpful' in 70% of instances. | Information transmission via telemedicine provides a clearer 'situational assessment' as a basis for decision support. It is possible that the favourable technical assessments have arised from positive attitude towards the system. Study conclusions were drawn from subjective situational assessment from the end-users of telemedicine. |
| (2011) | Diagnostic support | 6% |  |  |
|  | ECG indications | 8% |  |  |
|  | Treatment decisions | 7% | Data transfer was most useful in providing preliminary info to the hospital (no in-hospital patient outcomes were investigated) |  |
| Bergrath, S. | Diagostic support | 97.1% (23% under Trauma category) | TM assisted in diagnostic accuracy and medication administration. TM was also used for procedure delegation and in making receiving hospital decisions. | EMS disctricts not randomised therefore open to selection bias. |
| (2013) | Medication administration | 60% |  | Decisions being made and to be carried out reinforced by the presence of an 'expert'. |
|  | Receiving facility decision | 37% |  |  |
| Cho, S. J. | Voice consultation | Baseline Study group | No significant advantage when using telemedicine system or voice calls to describe patient status for medical decisions. Both telemedicine and voice calls were similarly useful in making a diagnosis and treatment decisions. | Telemedicine often requires additional effort and training, therefore, it would be most beneficial to embed as tool for routine emergency patient care. |
|  | - voice call | 117 13 *p* < 0.001 |  |  |
|  | - telemedicine | 0 69 |  | Technologically focused study and the non-significant preference for TM is based on 5-point Likert scale results. |
|  | On scene treatment |  | Fisher's exact results show more remote expert consultations being made prior to telemedicine implementation. This is possibly due to technical difficulties. |  |
|  | - voice call | 116 26 *p* < 0.001 |  | Nevertheless, this study highlights difficulties in translating telemedicine as methods of routine care. |
|  | - telemedicine | 0 41 |  |  |
|  | - connection failed | 1 15 |  |  |
| DeSoucy, E. | N/A | 8 Teleconsultations recorded | In 14.8% cases, telemedicine played an integral role in patient management. TM reviewed as one of the most valuable opportunity for improvement. | TM particularly useful for situations where there is a knowledge and experience gap, often complicated by austere setting. Simple telephone communication highly effective in providing access to remote experts -and ongoing treatment plan in prolonged field care. These findings should be interpreted in light of bias present in missing/incomplete surveys and retrospective recollection of events. |
|  |  |  |  |  |
|  |  |  | Most common consultants were surgeons and emergency physicians. |  |
|  |  |  |  |  |
|  |  |  |  |  |
| Dulou, R. | N/A | N/A | A retrospective report of TM use in 2/15 neurotrauma cases. Surgical guidance via TM allowed appropriate management without the deployment of MNSU. | Use of TM is descriptive and lack quantitative data. Therefore, it is difficult to determine the quality of evidence. The study, however, highlights the low cost, high impact usability of TM, especially in austere settings. |
|  |  |  |  |  |
|  |  |  |  |  |
| Kim, Y. K. |  | Study group *M* (SD) Control *M* (SD) | Higher frequency medical direction observed when using RTS TM system. Time to treatment was shortened for traumatic cases and more treatment oversight made available for advanced life support. | There may be differential effects for TM use for less severe patients vs. more severely injured/ill. Standardisation of emergency patient care is not yet available in South Korea. There are some concerns for data accuracy and credibility as the prehospital medical record does not appeared to be put into effect systematically. |
|  | Treatment time | 4.6 (3.6), 6.2 (6.6), *p* = 0.088 |  |  |
|  | Medical direction for treatment | 15 (8), 2 (0.3), *p* <0.05 |  |  |
|  |  |  | Receiving hospital facility decision support. |  |
|  | Ambulance diversion | 27 (14.4), 1 (0.1), *p* <0.01 |  |  |
|  |  |  |  |  |
| Yperzeele, L. | Intervention time | Study group10 minutes (IQR 7-13 minutes) vs. Controls M =36 minutes (IQR 29-51 minutes) NS | Accuracy for prehospital diagnosis via TM was high. Time to intervention was shortened in the study group without any significant differences in the type of treatment being delivered between the two groups. | Intervention time was recorded, however, the evaluation on the quality of medical care and its outcomes were not included in this feasibility study. |
|  |  |  |  |  |
|  |  |  |  |  |
|  | Inhospital and prehospital agreement on diagnosis | k = 0.98 and 0.92, *p* < 0.001 |  |  |
|  |  |  |  |  |
